# Learning from the End of the Public-Private Partnership for Lesotho’s National Referral Hospital Network

**DOI:** 10.5334/aogh.4377

**Published:** 2024-03-07

**Authors:** Chelsea M. McGuire, Jeanette L. Kaiser, Taryn Vian, Elizabeth Nkabane-Nkholongo, Tshema Nash, Brian W. Jack, Nancy A. Scott

**Affiliations:** 1 Department of Family Medicine, Boston Medical Center, Boston, MA, USA; 2 Lesotho Boston Health Alliance, Maseru, Lesotho; 3 Department of Global Health, Boston University School of Public Health, Boston, MA, USA; 4 School of Nursing and Health Professions, University of San Francisco, San Francisco, CA, USA

**Keywords:** heath systems research, public private partnerships, qualitative research, human resources, personnel, sub-Saharan Africa, low-and-middle-income countries

## Abstract

**Background:**

Public-private partnerships (PPP) are one strategy to finance and deliver healthcare in lower-resourced settings. Lesotho’s Queen ‘Mamohato Memorial Hospital Integrated Network (QMMH-IN) was sub-Saharan Africa’s first and largest integrated healthcare PPP.

**Objective:**

We assessed successes and challenges to performance of the QMMH-IN PPP.

**Methods:**

We conducted 26 semi-structured interviews among QMMH-IN executive leadership and staff in early 2020. Questions were guided by the WHO Health System Building Blocks Framework. We conducted a thematic analysis.

**Findings:**

Facilitators of performance included: 1) PPP leadership commitment to quality improvement supported by protocols, monitoring, and actions; 2) high levels of accountability and discipline; and 3) well-functioning infrastructure, core systems, workflows, and internal referral network. Barriers to performance included: 1) human resource management challenges and 2) broader health system and referral network limitations. Respondents anticipated the collapse of the PPP and suggested better investing in training incoming managerial staff, improving staffing, and expanding QMMH-IN’s role as a training facility.

**Conclusions:**

The PPP contract was terminated approximately five years before its anticipated end date; in mid-2021 the government of Lesotho assumed management of QMMH-IN. Going forward, the Lesotho government and others making strategic planning decisions should consider fostering a culture of quality improvement and accountability; ensuring sustained investments in human resource management; and allocating resources in a way that recognizes the interdependency of healthcare facilities and overall system strengthening. Contracts for integrated healthcare PPPs should be flexible to respond to changing external conditions and include provisions to invest in people as substantively as infrastructure, equipment, and core systems over the full length of the PPP. Healthcare PPPs, especially in lower-resource settings, should be developed with a strong understanding of their role in the broader health system and be implemented in conjunction with efforts to ensure and sustain adequate capacity and resources throughout the health system.

## Introduction

Ensuring access to high quality healthcare for people of all ages, in the setting of rising medical costs, is a complex challenge globally. Since the 1990s, governments in high- and, more recently, lower-resource settings have looked to healthcare public-private partnerships (PPPs) as a way to respond to this challenge [1,2]. PPPs are long-term, formalized contracts between the public and private sectors to provide services in a way that leverages the different strengths of each partner. Financial and operational risk is transferred to the private partner, who is accountable for defined outcomes such as quality metrics. The public partner retains ownership of the facility and equipment at the end of the contract [3]. Integrated PPPs add the delivery of clinical services within the private partner’s scope and are designed to create long-lasting improvements to the health sector via the combined investment in health capital and service provision [1]. A recently published review of hospital PPPs globally, however, suggests mixed results on hospital performance indicators and highlights important challenges to implementation including resources, trust and communication, and the policy context [4].

Sub-Saharan Africa’s first and largest integrated healthcare PPP was opened in 2010–11 as a partnership between the Ministry of Health of Lesotho and T’sepong, a consortium of Basotho and South African companies headed by Netcare, a large private hospital network based in South Africa [5]. A primary goal of this 18-year PPP agreement was to replace the 100-year-old national referral hospital, Queen Elizabeth II (QEII), through the design, construction, and operation of Queen ‘Mamohato Memorial Hospital (QMMH). Under the agreement the private partner also constructed a new Gateway ambulatory clinic on the hospital campus and renovated three filter clinics which provide outpatient primary care services and inpatient deliveries in the capital city of Maseru. QMMH and its four affiliated clinics are referred to collectively as the QMMH Integrated Network (QMMH-IN). This strategy of bundling both hospital and primary health care services within an integrated PPP is often referred to as the *Alzira* Model, named after the first hospital system in Spain, to pioneer this PPP strategy [6,7]. Details regarding the QMMH-IN PPP, its history, and prior evaluations are described elsewhere [1,5,8,9,10].

The PPP contract stipulated specific quality standards under which the network was to operate, ranging from waste management to personnel training metrics. If any standard was not met, the government could deduct a specific percentage from the monthly payment made to T’sepong. Two structures for monitoring these standards were employed. First, the consulting firm Turner & Townsend—referred to as “the independent monitor,” was engaged to monitor these standards quarterly [1]. Second, QMMH leadership was required to achieve and maintain accreditation by The Council for Health Service Accreditation of Southern Africa (COHSASA), a regulatory body for health services in southern Africa, which conducts monitoring and re-accreditation processes. QMMH facilities were first accredited in 2013 and they maintained accreditation status through February 2022 [11].

An evaluation comparing QEII performance data from 2006–7 to QMMH-IN performance data from 2012 found that the network increased quality of care, delivered more services, and produced better outcomes, including reduction in overall mortality by 41% in its first year [5]. Interviews conducted with the executive leadership team, department heads, and staff in 2013 identified perspectives on drivers of early hospital performance, including new clinical services, better infrastructure, human resource management changes, and innovation in management systems [12]. A follow-up evaluation based on clinical data collected in 2018 showed that despite challenges, QMMH-IN continued to provide high-level medical services, and generally maintained better patient outcomes and quality of care compared to the QEII baseline [10].

Over the course its lifespan, the PPP-managed QMMH-IN has been controversial [13,14]. Against the backdrop of increasing tension between the PPP partners with numerous disagreements under legal arbitration, the Government of Lesotho announced in early 2021 its termination of the PPP contract [15]. The hospital network leadership transitioned to government management mid-2021, approximately five years before the termination date in the original contract [16].

This paper explores the facilitators and barriers related to hospital performance under the PPP from the perspective of QMMH-IN leadership and staff (all employed by the private partner T’sepong) in early 2020. It aims (1) to generate ideas for consideration by the Government of Lesotho now responsible for direct oversight of QMMH-IN post-PPP and (2) to inform operations of health sector PPPs in other low-resource contexts.

## Methods

### Study design

This qualitative analysis is part of a larger evaluation of the QMMH-IN PPP in Lesotho, which included a baseline study in 2006–7 [8], an initial evaluation conducted in 2012–13 [5,9,12,17], and a follow-up evaluation in early 2020 [10]. Data for this analysis were collected from QMMH-IN leadership and staff between January–February 2020.

### Study setting

The Kingdom of Lesotho is a small, mountainous, lower-middle income country surrounded by South Africa, with a population of approximately 2.1 million. HIV remains the primary cause of death, followed closely by tuberculosis, and, as of 2017, life expectancy was 59 years for females and 50 years for males [18]. In 2020, an estimated 31% of the population lived under the international poverty line of $1.90 USD per day, with poverty highly concentrated in rural areas [19]. Just 29% of the population lives in urban parts of the country [20].

QMMH-IN is in Lesotho’s capital city of Maseru, the country’s major urban center. The three filter clinics operated under the QMMH-IN are spread throughout Maseru district and opened in May 2010. The ambulatory Gateway clinic and QMMH, which included an intensive care unit (ICU) and a neonatal intensive care unit (NICU), opened on the same hospital campus in October 2011. After QMMH replaced the old QEII in 2011, there was no separate publicly funded district hospital for Maseru. QEII reopened as an outpatient-only facility in 2014. As of 2018, QMMH was the only higher-level hospital in the area. It had 434 operational beds, with the three filter clinics adding a combined 24 additional short-term obstetric beds. Across the network, a total of 582 clinicians, including 295 registered nurses and 85 physicians were employed. In 2018 QMMH saw 24,796 admissions, with an average length of stay of 6.5 days and a bed occupancy rate of 99% [10].

### Data collection

We conducted semi-structured key informant interviews with the executive team, department heads, and staff working at QMMH-IN facilities. We purposively selected respondents using a maximum variation strategy to include clinical and non-clinical roles from all levels of the organization (ranging from executive management to nursing assistants to support staff) [21]. We included those who worked at the main QMMH hospital, as well as the Gateway clinic and each filter clinic. We sought to include at least one from each clinic and hospital department. The study team provided a list of positions to hospital administrators who assisted in the identification of individuals and scheduling of interviews. Interviewers met with hospital leaders before commencing the study to discuss study aims, procedures, and address concerns.

Interviews were conducted in-person between January–February 2020, and each lasted approximately one hour. Questions and prompts were semi-structured and based broadly on the WHO Health System Building Blocks Framework, with primary themes encompassing: service delivery, including infrastructure, provider network, management, safety and quality; hospital workforce; information; medical products and technologies, including the core systems to manage medications, equipment, and commodities; financing; and leadership/governance within the hospital network and government [22]. Respondents were interviewed once and were asked to compare current hospital network performance to what they had previously known, to explain the drivers of that performance, to anticipate challenges that could emerge at the end of PPP contract, and to offer suggestions for improvement. We adapted questions as interviews evolved. We audio recorded all interviews and took hand-written field notes. We captured key demographic information using SurveyCTO® Collect Software (Dobility, Cambridge, Massachusetts, USA) on encrypted tablets.

### Data management and analysis

Demographic data were summarized descriptively. Interviews were conducted in English and audio recordings were transcribed semi-verbatim into Microsoft® Word.

All transcripts were checked for accuracy by study team members against audio recording, but not returned to respondents for checking due to the evolving global emergency of COVID-19. Transcripts were imported into NVivo version 12.7.0 (QSR International, Burlington, Massachusetts, USA) for coding and analysis. We conducted a thematic analysis [23] using a combined inductive and deductive approach to coding, starting with broad codes from the interview guide and allowing room for new codes to emerge. Two researchers (CMM, JLK) coded all transcripts with the initial codebook. Given the heterogeneity of respondent demographics, we coded all interviews instead of stopping when saturation was reached within particular thematic areas. CMM then constructed a coding tree which contained emergent categories of barriers and facilitators and re-coded the data. Three authors (NAS, JLK, CMM) discussed and arrived at the major and minor themes. Memoing was used throughout this process to aid in reflexivity [24]. Further inputs (ENN, BWJ, TV) on identified themes informed the discussion.

Qualitative findings were organized by themes and sub-themes that emerged as facilitators and barriers of QMMH-IN performance, those related to respondents’ perceptions of QMMH’s future transition post-PPP, and their recommendations. Illustrative quotations were lightly edited for clarity and are displayed in tables, referenced by corresponding letters.

### Ethical issues

The Ministry of Health Research and Ethics Committee in Lesotho (Protocol 230-2019) and the Boston University Medical Campus Institutional Review Board (Protocol H-39448) approved the study. The interviewers (JLK, TN) were trained in research ethics, the overarching study, and the specific interview guides. After introducing themselves, interviewers shared an information sheet with each respondent about the study and described study objectives, potential risks, benefits, and guarantees of confidentiality, addressing respondent questions and concerns. Verbal informed consent was obtained for each interview and audio recording. Respondents skipped questions they were not comfortable answering. No individual refused to participate or withdrew. Interviews were conducted in a private office, with only the interviewer and respondent present. Data are presented in aggregate form and anonymized to ensure responses remain confidential.

## Results

### Demographic characteristics

The mean age among respondents was 43.3 years (standard deviation [SD] 8.4 years) and just over half were female. Twenty-one respondents (80.8%) worked at QMMH, while five worked at the network clinics. Sixteen (62.5%) held clinical roles. Fifteen (57.7%) were in higher management positions, and respondents had been employed in the PPP for an average of 7.7 years (SD 2.5 years) (Table 1).

**Table 1:** Demographic Characteristics of Queen ‘Mamohato Memorial Hospital Integrated Network Interview Respondents.


CHARACTERISTIC VARIABLES	INTERVIEW RESPONDENTS (n = 26)

Female, n (%)	15 (57.7%)

Age (years), mean (SD)	43.3 (8.4)

Organization, n (%)	

*QMMH*	21 (80.8%)

*Network clinics* ^a^	5 (19.2%)

In clinical role ^b^, n (%)	16 (62.5%)

In higher management position ^c^, n (%)	15 (57.7%)

Years in current position, mean (SD)	4.1 (2.6)

Years employed in PPP, mean (SD)	7.7 (2.5)


SD = Standard deviation; PPP = Public-private partnership; QMMH = Queen ‘Mamohatu Memorial Hospital. ^a^ Network clinics included the Gateway and three filter clinics. ^b^ Clinical roles include physicians, nurses, and pharmacists.

### Facilitators of QMMH-IN performance

Three major themes emerged as facilitators of QMMH-IN performance: (1) a commitment to quality improvement supported by protocols, monitoring, and actions; (2) high levels of accountability and discipline; and (3) well-functioning infrastructure, core systems, workflows, and internal referral network (Table 2).

**Table 2:** Illustrative quotes from respondents on perceived facilitators of QMMH performance.


THEME	SUB-THEME	ILLUSTRATIVE QUOTES

**I. Protocols, monitoring, and quality improvement**	**1. Use of clinical protocols led to higher quality care**	**a)** “Patient outcomes—they’re incredible. When you have standard operating procedures, you basically have a map of how you are going to care for patients.” – QMMH staff, clinical roleb) **b)** “The most important change is quality care (…) [By] having guidelines and policies, one knows what to do and how [to do it]. Hence, in the end, we have quality care.” – QMMH higher management, clinical role

	**2. Routine internal and external monitoring as driver of performance**	**c)** “We monitor the performance of the clinicians and the nurses. We do clinical audits to see whether they are conforming to the protocols and the clinical pathways (…) [We] come up with quality improvement projects that will improve from where we are and eliminate gaps that were identified.” – QMMH higher management, clinical roled) **d)** “For every department we do a risk assessment, risk rating, and monitoring (…) They report the near misses, the incidents, the adverse events, and the sentinel events. (…) The policy is that if there has been any sentinel or adverse event, within five days we should have done a root cause analysis and, within 20 days, advise the management on what to do.” – QMMH staff, non-clinical rolee) **e)** “It’s all about meeting goals and having this independent monitor to come and check that things are being dealt in accordance to what has been requested by the contract. It puts everyone on their toes (…) There is an evaluator [who comes to] check your work functions, evaluate [you] and give you a penalty if you are not performing well. This positive change in performance [is] because there is an evaluation and monitoring process.” – QMMH higher management, non-clinical rolef) **f)** “Because we try to adhere to COHSASA, our standards are much higher. We get evaluated so I think our services are a lot better than the previous hospital.” – QMMH higher management, non-clinical role

	**3. A dedicated office of quality and risk led staff actions to address gaps and improve quality**	**g)** “QMMH doesn’t joke, if there is a loss of life we have to sit down (…) to see what went wrong. The fish bone [diagram] is really scrutinized. And the people [then] know that, yes, I could have changed the outcome. And next time we [see] this case, I am not going to miss that. (…) [It is] the most important change.” – Clinic higher management, clinical role **h)** “[If] we meet certain obstacles, we come up with quality improvement projects (…) because we have a quality office here and they really ensure that in all aspects everything is on point.” – QMMH staff, clinical role

**II. Accountability and discipline**	**4. Accountability started with clear roles, responsibilities, and polices**	**i)** “What happens is on day-to-day, employees know what is expected of them because they have their job descriptions.” – QMMH higher management, non-clinical rolej) **j)** “We know what time we have to be in at work and what time to knockoff. Things with the PPP have been going so well, compared to where I was working before, because I would leave [early] at 1: 00pm and nobody will ask me anything. Here I know that even if nobody asks me anything, there are things I should follow. I have to come at a specific time. I have to do [specific work]. There are guidelines, policies.” – QMMH higher management, clinical role

	**5. Individual performance assessed using a balanced scorecard**	**k)** “Before I can assess the employee, we need to fill out the balance scorecard so that they can know which issues I am going to assess them on (…) [The balanced scorecards] have helped in a positive way. People don’t want to make any mistakes (…) [and] if they have made mistakes (…) we can rectify.” – QMMH higher management, non-clinical rolel) **l)** “Even me, I have a balance scorecard (…) my goals are this: to decrease morbidity in the department, to decrease neonatal deaths. Every quarter I have to translate to where we left and where we are in this quarter. Do we see a decrease? And then be accountable to say what measures am I going to implement in the department to ensure that we achieve what reflected in the score card. (…) It is really well structured. Hence why a doctor who is not used to that gets frustrated. They get to a point where they are monitored, monthly, weekly, quarterly to say: ‘but you are not performing.’ So it really helps to keep the performance standards.” – QMMH higher management, clinical role

	**6. Disciplinary actions taken to help ensure quality**	**m)** “If you don’t perform or you are found guilty of mismanagement of patients, steps are taken about such individuals. Some of them had to be sacked because of those reasons, and you hardly find that happening in a government setup. These things make people stand on their toes (…) I think this is [a] positive change.” – QMMH higher management, clinical rolen) **n)** “A receipt is given to every single service that has been done. Stickers and receipts are verified. We had discrepancies where we found that the clinics, their payments were done but were not dropped in the safe. And a whole lot of employees were fired and disciplinary [meetings] were held (…) They are very particular in checking. Yes, there will be loopholes, [but] when we find them, we do something about it.” – QMMH staff, non-clinical role

	**7. Accountability systems, such as biometric clocking and employee numbers, promoted professionalism and discipline which led to increased quality**	**o)** “You account for every minute of your time. We do a biometric clocking system. Those are things that don’t exist in the [public] health sector. This culture in the private sector makes a difference in terms of outputs.” – QMMH higher management, non-clinical rolep) **p)** “The cashiers use [an] employment number which they enter in the [accounts] system. During lunch time when they are being relieved, they have to balance their money, drop it in the safe, and then go for lunch. Whoever is coming in, she also is going to use her employment number so there is not going to be any mixed ups.” – QMMH higher management, non-clinical role **q)** “We are disciplined because of the kind of management we have. You won’t arrive at work at 10:00 when you are supposed to arrive at 7:00. That means services are going to run on time. People don’t steal the medications from the pharmacy (…) Even as staff [when I am sick], I know I have to consult with a doctor, pay the 15 Rand, get my medication. [This] is not the case in those other facilities. (…) This is why I said I can advocate for another PPP facility.” – QMMH staff, clinical roler) **r)** “They [management] has their eyes on the staff. They know what is happening where. Who is performing and who is not performing. We are [more] closely monitored than at many other places. I think that is the good thing that is happening. [However], I know there is also some dissatisfaction in certain ways [with this close monitoring].” – QMMH higher management, clinical role

**III. Infrastructure, core systems, workflows, and internal referral network**	**8. High quality and maintenance of physical infrastructure including facilities and equipment**	**s)** “It’s the infrastructure. It’s new and improved. People have access to a well-maintained facility.” – QMMH higher management, non-clinical rolet) **t)** “In the wards there are cubicles, there are curtains where the patients are seen and you draw them. All our consultation rooms are private. (…) Patients are given the privacy they need.” – QMMH higher management, clinical roleu) **u)** “Having access to clinical services that didn’t exist before, that’s a big deal for me. Because for instance, the MRI machine didn’t exist before, it [allows for] better diagnosis.” – QMMH higher management, non-clinical rolev) **v)** “If I report that my ultrasound machine is out of function, it’s going to be repaired immediately. [Conversely] sometimes at Queen Elizabeth II, six months down the line you find the X-ray machine is still broken (…) So patient satisfaction goes with that, [at QMMH] you will not have to come several times and still find [equipment that is] not functional and be returned.” – QMMH higher management, clinical role

	**9. Strong core systems, including pharmacy and laboratory, and hospital workflows**	**w)** “The difference between us and the government is that we don’t do long term procurement. We buy more regularly (…) so we don’t necessarily experience long-term stockouts [at QMMH]. If it is an essential medication, it is categorized into E, VI, HV - Essential, Very Important, and High Volume. You make your assessment. If it’s something you feel a week is too long, you can’t survive, [but that medication isn’t available from the government stock,] we immediately outsource it from elsewhere.” – QMMH higher management, non-clinical rolex) **x)** “[QMMH is] incredible compared to Queen Elizabeth II, where you would have to run around and go look if you have got [laboratory] results [back]. Here its digital (…) An ABG (arterial blood gas) you are going to get results in five minutes time. So when it comes to the lab, it’s really effective. We even have a way of tracking the specimens.” – QMMH staff, clinical role

	**10. Efficient internal referrals and collaboration across network**	**y)** “The [internal] system of a collaboration between the [filter] clinics and hospital is perfect. Because (…) there is all this collaboration, at a high level with the heads of departments [passing] down all the information to the [staff] of the department. And the referral system, it’s really well strategized (…) Among the audits I do in a regular basis, I [evaluate] to find out how many patients were referred. What was the outcome? What was the condition of the patient? Was it a delay in referring [the] patient? So all this we monitor on a regular basis. There has been a tremendous improvement.” – Clinic higher management, clinical rolez) **z)** “The network really helps because I don’t see one [facility] working alone in isolation. An example [is] exchanging of drugs, as in borrowing, maybe those that are due to expire. (…) They [patients] will be put on a particular medication that really improves their lives. So when they [patients] go back to their original places, they need the same medications, and sometimes those people there don’t have that medicine, it is only at the referral hospital. So, we said okay, the recipient hospital will borrow this much for the patient until they get theirs.” – QMMH higher management, clinical role


COHSASA = Council for Health Service Accreditation of Southern Africa; QMMH = Queen ‘Mamohato Memorial Hospital. Note: Ellipses indicate removed text to shorten quotes, while preserving meaning. Square brackets contain text added by the authors to facilitate comprehension.

#### Protocols, monitoring, and quality improvement

The first driver of QMMH-IN performance was a dedication to quality improvement (QI) facilitated by use of clinical protocols, internal and external monitoring, and steps taken to address quality gaps.

*Clinical protocols:* There was broad agreement that the availability and routine use of specific clinical care guidelines and protocols, sometimes referred to as standard operating procedures for care, were key drivers of quality clinical care. Following evidence-based practices was perceived to lead to improved patient outcomes and thus network performance (Table 2, quotes a–b).

*Internal and external monitoring:* Respondents emphasized that they perceived monitoring at QMMH-IN to be regular and consistent. A variety of internal monitoring structures were described as facilitating performance. For example, respondents’ spoke about routine clinical audits to identify opportunities for targeted training, updates to the clinical protocols, or quality improvement projects (Table 2, quotes c–d). Monitoring also included risk assessments and ratings of departments based on the frequency of adverse events, near misses, and sentinel events (Table 2, quote d). Respondents discussed regular evaluation visits by the external Independent Monitor who assessed everything from hand-hygiene to waste management, as well as the pressure to maintain COHSASA accreditation standards (Table 2, quotes e–f).

*Taking action to improve quality:* Respondents reported that the gaps identified through monitoring activities resulted in actions that, in turn, improved performance. They emphasized how quality improvement and risk mitigation are taken seriously (Table 2, quote g). Clinical and non-clinical respondents alike discussed how they regularly applied tools such as root cause analysis to act on identified gaps and make corrective changes to avoid future mistakes. A dedicated office of quality and risk led these actions and demonstrated the network’s commitment to QI implementation (Table 2, quote h).

#### Accountability and discipline

Respondents explained that the focus on QI at QMMH-IN translated to policies, tools, and other factors that promoted a high level of individual accountability and discipline.

*Clear roles and policies:* Respondents reported to have clarity regarding their roles, performance expectations, and the policies to follow. This, in turn, promoted individual accountability and behaviors such as arriving to work on time and completing all expected tasks (Table 2, quotes i-j).

*Balanced scorecards:* Employees’ performance was regularly monitored by using a tool called the balanced scorecard, which provided very specific metrics based on the job description of the employee (Table 2, quote k). Supervisors used balanced scorecards to develop individualized action plans to address deficiencies. Respondents noted that the metrics tracked on their own balanced scorecard related to broader QI goals, such as reducing mortality rates (Table 2, quote i).

*Disciplinary action:* Many respondents stressed that disciplinary action was taken when needed, and employees were let go if their performance was consistently poor. Clinicians were assessed on their skills and knowledge before being offered a contract renewal. Respondents said this was in contrast with the public facilities where it was difficult to terminate employees (Table 2, quotes m-n).

*Accountability systems to foster professionalism:* QMMH-IN used a variety of systems that promoted accountability, such as biometric clocking (Table 2, quote o). Another example was the use of employee numbers to record who had logged into a system that tracks movement of money from a cashier to the safe (Table 2, quote p). Respondents generally described these systems as powerful tools that increased discipline and professionalism. They perceived this as translating to increased punctuality, decreased theft, and overall improved performance (Table 2, quote q). A few respondents reported some employees were dissatisfied with the intense oversight and accountability systems (Table 2, quotes r, l).

#### Infrastructure, core systems, workflows, and internal referral network

The high quality and functionality of physical infrastructure, core hospital systems and workflows, and internal network collaboration were also perceived to facilitate QMMN-IN performance.

*Facilities, equipment, and their maintenance:* Nearly all respondents spoke highly of the QMMH-IN physical infrastructure, describing it as well-maintained, clean, and well-designed to ensure patient privacy (Table 2, quotes s–t). This was perceived to improve the well-being of not only patients, but also employees, who work better in a comfortable and safe environment.

Respondents generally felt necessary equipment was available and well-maintained. With a few exceptions, mostly related to delays in receiving parts from South Africa, respondents were pleased with the company contracted to provide, maintain, and repair the medical equipment, and felt it improved clinical performance and patient satisfaction (Table 2, quote u–v).

*Core systems and workflows:* When comparing to public facilities, respondents generally felt the core systems such as pharmacy and laboratory services facilitated performance, particularly emphasizing the availability of medications. Respondents felt that procuring supplies weekly and outsourcing when needed decreased pharmacy stock-outs (Table 2, quote w). Respondents felt this improved patient outcomes and shortened hospital stays.

Respondents had mixed opinions of the laboratory system, citing a recent change from sub-contracted lab services to the lab being managed in-house. Despite challenges with the new, in-house lab, most respondents still considered this core system a facilitator, highlighting the quick turnaround times and ability to track specimens digitally from collection through receipt of results (Table 2, quote x).

Other examples of specific workflows perceived to improve QMMN-IN performance included individualized delivery of medications to patients on the wards, stock monitoring, and computerized maintenance schedules for equipment.

*Internal referrals and network collaboration:* The system for referring patients from the filter clinics to the QMMH hospital also emerged as a perceived facilitator. Respondents felt that referral audits and regular communication through academic meetings between clinicians at the various network facilities improved the timeliness and quality of referrals. Collaboration across the network also allowed for other efficiencies that were perceived to improve patient care, such as the ability to share medications between sites (Table 2, quotes y–z).

### Barriers to QMMH-IN performance

Two major themes emerged as barriers to QMMH-IN performance: (1) challenges within human resource management; and (2) broader health system and referral network limitations (Table 3).

**Table 3:** Illustrative quotes from respondents on perceived on barriers QMMH-IN performance.


THEME	SUB-THEME	ILLUSTRATIVE QUOTES

**I. Human Resource Management Challenges**	**1. Poor salaries and working conditions impact on recruitment and retention**	**a)** “A big factor driving change in performance is salary. The biggest tool you have in a hospital is the staff. It’s very difficult to keep the staff if other ministerial departments pay more.” – QMMH higher management, non-clinical role **b)** “As for these shifts we are working, most people like them. They are used to working 12 hours shifts, while in government people are working 8 [or] 9-hour shifts. They say they are fine with the shifts. They only complain about money.” – QMMH staff, clinical role **c)** “For a tertiary hospital we should have people who are more experienced, but that’s the opposite because of issues of salaries we usually get. So [that is] quite challenging. We have newly graduated nurses coming to work here (…) we need people with skills that are mature.” QMMH higher management, clinical role

	**2. Limited staff and clinical specialists**	**d)** “We feel we are understaffed (…) the nurse-patient ratio is one nurse to ten patients. But given the work that we are doing here and the quality of work that we provide to our patients (…) [and] the type of patients that we treat here - those that could not be treated anywhere else (…) we feel we are severely understaffed.” – QMMH staff, clinical role **e)** “There are no ICU specialists; [nor] emergency specialist.” – QMMH higher management, clinical role

	**3. Insufficient focus on training**	**f)** “The hospital, from what we understood at the beginning, was to reduce referrals and to train people. Train doctors, specifically local doctors, so that they then care for most of the patients locally. That is not happening as expected (…) There has to be a lot more effort with regards to developing the doctors (…) We don’t have a well-organized training program which should be there.” – QMMH higher management, clinical role **g)** “Queen Mamohato [is] not really empowering the [rest of the health] system (…) [The] job description talks about external capacitation, [via] internal and external interaction. But most of the time, the current system is not flexible enough to allow external participation.” – QMMH higher management, clinical role

**II. Limitations of Structure and Function of larger Health System and Referral Network**	**4. Perceived lack of capacity at district hospitals increasing patient load at QMMH**	**i)** “QMMH is a symptom of what is broken in the system. Patients are flooding QMMH because it’s the only place that they feel they can get help. So QMMH ended up doubling [it’s expected] numbers of patients (…) some of these patients, maybe caesarian section for fetal distress, could have been done at a district hospital but they say they don’t have oxygen, so patients are referred (…) The entire health system needs to be strengthened.” – Clinic staff, clinical role **j)** “There are no resources in government institutions. (…) You tell [patients] to go to a hospital [and] they cry, they don’t want to go (…) the entire service delivery in government institutions is not functioning as one would expect it to (…) You walk into a [public] clinic and maybe there is only one nurse working there. It’s very difficult. People queue for very long time. I think it’s easier for people to trust Queen ‘Mamahato rather than the government institutions.” – QMMH staff, clinical role

	**5. Inappropriate referrals from outside facilities leading to QMMH not functioning at intended level**	**k)** “Patients will be referred here [at QMMH] who don’t need to be here (…) there was no linen at the district hospitals so they could not do a caesarian-section (…) We can have a better functioning system. [That is] our challenge. We get unnecessary referrals.” – QMMH higher management, clinical role **l)** “[The existence of QMMH] has decreased the quality of care provided by the district hospitals because now if you go to the district hospital, people will just refer something that they could have treated locally. Refer. But we don’t see that from the filter clinics (…) I think [the presence of QMMH] has taken away the clinical skills of people [working in the districts].” – QMMH higher management, clinical role **m)** “Patients are coming late, they are referred late, and therefore the outcome is bad. So we can’t blame the hospital [QMMH] for the poor outcome of the patients. (…) Providers will waste time in the in private clinics up until it’s late, they will send a patient to QMMH when it’s late.” – Clinic higher management, clinical role **n)** “The closing of Queen Elizabeth II caused a lot of havoc in the whole [health] system, because Maseru did not have a district hospital. Only recently Queen Elizabeth II was opened [again] and only the outpatient department. Now you find that QMMH has literally becomes the district hospital [and] the referral hospital. (…) Service provision has been affected by trying to share resources between managing primary cases and high-risk cases. And the bulk of patients that come here are primary care patients.” – QMMH higher management, clinical role


ICU = Intensive Care Unit; QMMH = Queen ‘Mamohato Memorial Hospital. Note: Ellipses indicate removed text to shorten quotes, while preserving meaning. Square brackets contain text added by the authors to facilitate comprehension.

#### Human resource management

Recruitment and retention of adequate levels of staff and specialty clinicians were described as barriers to performance.

*Recruitment and retention:* Respondents felt that QMMH-IN performance was limited by its inability to recruit and retain experienced employees, primarily due to inadequate compensation. Respondents from across departments expressed dissatisfaction with their salaries, in relation to the work they are doing, and in comparison to salaries thought to be offered at governmental health facilities (Table 3, quote a), non-governmental organizations, and private mining companies. One respondent specifically described that while most did not mind working longer hours than their government counterparts, they wanted appropriate compensation (Table 3, quote b).

Respondents felt inadequate compensation led to poor morale and high employee turnover in all departments, explaining QMMH-IN struggled to recruit experienced nurses and physicians, and then to keep them after they were trained (Table 2, quote c). They perceived this to affect patient outcomes since newer clinicians often do not have the necessary experience to provide the level of care expected at a referral hospital.

*Staffing and specialists:* Some respondents noted that staffing ratios and number of specialists had improved within QMMH-IN compared to before the PPP and to other hospitals. However, others expressed that these figures were still insufficient and limited network performance, noting specifically a lack of ICU and Emergency Medicine specialists (Table 3, quotes d–e).

*Training:* While some described the training offered by QMMH-IN positively, most respondents described a lack of training opportunities that limited employee potential. Some respondents felt the lack of emphasis on training and professional development for clinicians made QMMH-IN fall short of its contractual mandate (Table 3, quote f). Another respondent discussed the limited focus on capacity building in the broader health system (Table 3, quote g).

#### Lesotho health system and referral network

Limitations in structure and function of the district health system and referral network posed important barriers to QMMH’s performance and ability to serve as the national referral hospital.

*District health system capacity:* A common perspective among respondents was that Lesotho’s district health system lacked capacity in terms of needed equipment, medications, and staff to appropriately mange patients. This resulted in a “flooding” of patients who should have been managed at the district level coming to QMMH instead (Table 3, quote i). Respondents described patients as preferring to seek care at QMMH-IN due to a lack of capacity at the district level (Table 3, quote j).

*Inappropriate referrals:* Respondents also described receiving referrals who are not medically complex and should not be at QMMH (Table 3, quotes i, k). Respondents suggest this pattern has contributed to a decline in skills and further reduction of capacity at the districts (Table 3, quote l). Other respondents perceived that some patients are referred too late and arrive with advanced disease, resulting in poor clinical outcomes (Table 3, quote m). The use of resources to manage inappropriately referred cases at QMMH was perceived to have diverted resources away from cases that required specialty care, and overall leading to QMMH not performing at the level of a national referral hospital (Table 3, quote n).

### Perspectives and recommendations on transition of QMMH-IN Post-PPP

At the time of the interviews, the early end of the PPP contract had not yet been announced and a transition in QMMH-IN management was anticipated to happen in approximately six years. Respondents were asked what they anticipated to happen at the time of this transition and to provide recommendations for improvement now and at the time of transition. Table 4 outline the themes and illustrative quotes from their responses.

**Table 4:** Illustrative quotes from respondent perspectives on and recommendations for transition of QMMH-IN after PPP ends.


THEME	SUB-THEME	ILLUSTRATIVE QUOTES

**Perspectives on transition of QMMH-IN after PPP**	**1. Concern over management transition**	**a)** “Just see how the government hospitals are managed. How the services are in the government hospital. So if the hospital is given back to the same management, all of this [at QMMH] will fall. That is my personal opinion.” – QMMH higher management, clinical role

	**2. Insufficient preparation for transition**	**b)** “I don’t think this infection control office, quality office and all of that will be functional. I think as soon as government takes over, things are going to deteriorate. Because if they were copying what QMMH is doing now, and trying to implement it at the district hospital, I would think they will be able to manage the hospital by then. But seeing that they have not even started [trying to learn what QMMH is doing], I am not convinced they will run this hospital to a level that it is at now.” – QMMH staff, clinical role

	**3. Poor receipt of quality standards at government facilities**	**c)** “[There was a] pilot of the COHSASA standards [at] three institutions, [including] Christian Health Association of Lesotho institutions and government. The assessors of COHSASA said: [for] government institutions – this is an impossible task. [The institutions said] we will not be bothered; we don’t want this thing of yours. You can go away with your standards (…) You see, they [the government-run hospitals] are untouchable.” – QMMH staff, non-clinical role

**II. Recommendations for transition of QMMH-IN post-PPP**	**4. Keep what works: culture of quality improvement, evidence-based practice, and well-functioning network systems**	**d)** “Keep on doing the good things that you are doing. Learn about the new things that are coming up, and make sure that you improve and adhere to the new research and evidence-based practice.” – QMMH higher management, clinical role **e)** “In regard [to the] availability of medications. We already [have] the strategies of how we make sure things are available (…) which should be maintained (…) Maintaining the camera-surveillance system will also save the budget of this country. I am even suggesting it can go to other areas [other hospitals] (…) I am able to see what our filter clinics have while I am sitting here. This can be an improvement for the country to have a centralized area with computers that is it able to locate where everything is.” – QMMH higher management, clinical role **f)** “Botle, they’re a private [maintenance] company. If I were [the government], I would keep them (…) because they know everything. They have the floor plans, everything. So if you have somebody else they are going to [have to use] the first year try to figure out [what to do].” – QMMH staff, non-clinical position

	**5. Invest in management succession training**	**g)** “The government must start by preparing management [staff] that will take care of this hospital (…) So let’s train people so that when take over here they know what to do.” – QMMH higher management, clinical role **h)** “Make sure that you have at least a specialist in every department. Two in the big departments; have two surgeons, two physicians, two obstetricians, two pediatricians, two ophthalmology, maybe one ENT, dental one. If you have spread out the skills like that, you develop in every area. If that training program was there (…) by the time the government takes over, you have people who can head those departments.” – QMMH higher management, clinical role **i)** “Other clinics around us are referring patients to us and then the staff start feeling that they are underpaid. And yet they have to do more work than their colleagues who are working in smaller health centres, but they are making more money than they are. It’s an issue of creating better job satisfaction [through better pay].” – Clinic staff, clinical role

	**6. Prioritize achieving adequate staffing levels and numbers of trained specialists by investing in employees through training, advancement opportunities, and appropriate salaries**	**j)** “You’re handing this over a few years from now. There are contractual obligations that when you hand over equipment should be within its usable lifespan…I say start offering more trainings to people (…) The building, structural equipment should be intact when handed over. What about staff? Are they going to be here?” – QMMH staff, non-clinical role **k)** “Do projects that are geared towards employees’ empowerment. We have people who need to go to school; (…) sponsor them to do part-time studies. That would help all other things, because if our human resources are not doing well then (…) dissatisfaction will arise. But if projects are geared towards their [staff] improvement and wellbeing, that would motivate staff. If you have motivated staff, then the output is good.” – QMMH staff, non-clinical role

	**7. Have QMMH be a training facility for the districts**	**l)** “Doctors come from all over the world to Lesotho (…) Before they get dispersed into the other districts, they would sit at Queen Elizabeth II for six months for training. Which was excellent, because you know what they know, you know what they do not know, you put emphasis on developing them. (…) you have self-sufficient hospitals because you have appropriate doctors for that facility, but now we don’t have that. I don’t know [if] QMMH can help the country with that.” – QMMH higher management, clinical role

	**8. Invest in system strengthening and referral network improvements**	**m)** “[We need] a functioning health system and a good referral system so that people are referred [only] if they qualify (…) As it is now, people who come [to QMMH] are people with basic things that need to be attended to at the district health hospitals.” – QMMH higher management, clinical role **n)** “I think as a country as a whole, we need to try to have a network like [QMMH-IN] Where, there are levels of care: primary, secondary, tertiary level of care. We don’t want patients coming straight to casualty with cough and diarrhea whilst they could have been attended to at the clinic level.” – QMMH higher management, clinical role


COHSASA = Council for Health Service Accreditation of Southern Africa; ICU = Intensive Care Unit; PPP = Public-private partnership; QMMH = Queen ‘Mamohatu Memorial Hospital; ENT = ear nose and throat physician. Note: Ellipses indicate removed text to shorten quotes, while preserving meaning. Square brackets contain text added by the authors to facilitate comprehension.

Respondents expressed concern over the anticipated transition of QMMH-IN to government management and many predicted a decline in hospital performance (Table 4, quote a). Some felt frustrated that, at the time of the interviews, they did not feel the government was actively planning for the transition, nor learning from what worked well at QMMH-IN in order to prepare for it (Table 4, quote b). One respondent detailed an earlier unsuccessful attempt to pilot the implementation of COHSASA quality standards at government hospitals to illustrate their concern over the management transition of QMMH-IN (Table 4, quote c).

#### Recommendations for transition

Respondent-generated suggestions specific to the transition of QMMH-IN to the governmental management are summarized below.

The first recommendation was to keep, reinforce, and expand upon what works well now, including culture of quality improvement, a focus on evidence-based practice, and well-functioning medication supply, equipment maintenance, and communication systems within the network. To help achieve this, respondents suggested continuing to work with specific private contractors, especially in the early years of transition, to retain institutional knowledge and continue benefiting from well-established workflows (Table 4, quotes d–f). Respondents recommended the government invest in training managerial staff who will assume operations of the QMMN-IN to ensure they are adequately prepared for the transition (Table 4, quote g). Additionally, it was recommended to improve staffing levels and number of specialists by focusing on job satisfaction, recruitment, and retention. Specifically, respondents emphasized the need to offer employees training and career advancement opportunities and ensure salaries are competitive (Table 4, quotes h–k).

Furthermore, respondents suggested the government consider designating QMMH as a training facility, similar to how QEII previously functioned, to help maintain a standard of care for health providers working across the health system (Table 4, quote l). This could be part of an overall effort to invest in much needed strengthening the greater Lesotho health system. Lastly, respondents recommended improving the referral system between outside facilities and QMMH so that QMMH can function as a referral hospital as intended. The QMMH-IN internal referral network could then serve as a model for needed changes to the external referral network (Table 4, quotes m–n; Table 3 quote i).

## Discussion

QMMH-IN, as sub-Saharan Africa’s first and largest integrated PPP, is an important global experiment of how to fund and deliver high-quality medical care in lower-resource settings. Through this research, we explored insights and lessons learned from the insider perspective of QMMH-IN employees just prior to the early termination of the PPP contract. Facilitators of QMMH-IN performance included a focus on quality improvement; accountability and discipline; and well-functioning infrastructure, core systems, and workflows. Many of these elements were also observed in the 2012–2013 evaluation of the network. Major barriers were related to human resource management and QMMH’s relationship to the greater health system, both of which had newly emerged since the 2012–2013 evaluation [12]. At the time of data collection, although PPP partners were actively attempting to renegotiate the QMMN-IN contract, there was no known plan for an early termination of the PPP and transition in management. When asked about the anticipated contract end in six years, respondents expressed concern about the government’s capacity to manage QMMH-IN and maintain the level of hospital performance observed under the PPP and provided key recommendations for consideration around the time of transition. Perspectives on the early PPP contact termination are explored elsewhere [25]. As of mid-2021, the Government of Lesotho had assumed management responsibility of QMMH-IN. These findings may be relevant when making strategic planning decisions going forward.

We identified a strongly embedded culture of QI and accountability at QMMH-IN as a primary facilitator of the network’s performance. Sustaining this culture will therefore be important for the continued success of QMMH-IN. Doing so requires an intentional and systematic strategy [26] that includes each of the foundational elements of QI culture, namely: leadership commitment, empowered employees, customer focus, a collaborative environment, and the maintenance of an explicit QI infrastructure, including continuous data collection and monitoring by dedicated employees [27]. Employee empowerment, which was identified as a facilitator in the earlier evaluation of the network [12,17], appears to have declined in our 2020 evaluation in the setting of poor morale, and will need to be a focus of future efforts if this overall culture of QI and accountability is to be sustained. The continued use of the balanced scorecard or similar tool may be helpful to retain, as respondents in this study perceived it as motivating for employees. Other research supports the usefulness of balanced scorecards in linking individual roles and organizational performance goals [28] and stimulating QI culture [29]. Additionally, taking holistic view that includes sustaining workflows that ensure reliable procurement, quality assurance of needed medical products and technologies, and maintenance of the physical infrastructure and equipment cannot be overemphasized [30]. Each of these pieces of the system is interdependent and overall builds to a what has been previously described at QMMN-IN as a “reliable work environment [12].”

The primary barriers internal to QMMH-IN perceived to limit performance were related to human resource management, with low salaries in comparison to outside facilities and lack of investment in training opportunities driving poor recruitment and retention of experienced employees. Unanticipated wage increases of approximately 80% for government hospital workers announced in 2013 meant that QMMH-IN employees went from being paid 3–54% more than their governmental counterparts (depending on job category) to being paid less [9], partly due to a failure to renegotiate the PPP contract to take into account increased wage expenses. Financial strains on the network and the poor payment history by the Government of Lesotho [31] may have contributed to a decreased emphasis on training opportunities over time. It is widely recognized that the management, training, and retention of highly skilled medical staff is a key factor in health system performance and demands significant effort to sustain [32].

The main identified performance barrier external to QMMN-IN was the perception that lack of resources elsewhere in the health system was resulting in inappropriate referrals of low-complexity patients. This was also new in our analysis relative to the 2012–2013 QMMH evaluation [12], and is illustrative of how a PPP may influence supply and demand for healthcare over time. This barrier must be considered within the context of having no Maseru district hospital at the time, which undoubtedly exacerbated the number of patients presenting to QMMH-IN facilities [1]. Additionally, similar to what was seen in the comparative evaluation of PPP-run versus publicly run district hospitals in India [33], respondents in our study reported that patients themselves were also driving the “flooding” of QMMN-IN due to perceived higher quality care. PPP design and implementation must consider not only the hospital network itself, but also the broader healthcare system, as neither operates in isolation and effective national healthcare delivery strategies must consider care across the continuum[3,8].

### Considerations for Implementers and Management of PPPs

Below we summarize three considerations from these findings that may be of use to others interested in implementing healthcare PPPs, especially those in lower-resource contexts.

1. Build, embed and sustain a culture of QI and accountability that is supported by core systems, clinical and administrative workflows, and consistently maintained facility infrastructure. Our results suggest that this culture was a key driver of QMMH-IN performance and that maintaining it would be important for the continued performance of the hospital network. Some specific considerations include intensive succession planning for any new network managers, maintaining structures such as the Office of Quality and Risk, and continuing the use of accountability tools such as balanced scorecards. A follow-up study post-PPP could help to understand what happened with the transition and explore the degree to which this culture and the overall reliable work environment were sustained. This is responsive to identified gaps in the PPP literature including a lack of “whole life cycle” evaluations [34] and the need for evidence regarding transitions of healthcare PPPs [35].
2. *Invest in people as substantively as in infrastructure, equipment, and core systems*. Employee morale is crucial to hospital network performance; we observed a perceived lack of investment in employees over time, including lower pay than government counterparts and insufficient training opportunities. QMMN-IN did not respond to external changes to governmental healthcare worker wages, potentially to the detriment of network performance. Integrative PPP agreements should be flexible enough to allow for adjustments to external conditions in order to continue to prioritize adequate human resource investment over time. The importance of contract flexibility and responsiveness to changing external factors has been echoed throughout the PPP literature [1,34,36].
3. *Invest in broader health system strengthening*. Without adequate, concurrent investment in the health systems at the district and clinic level, QMMH-IN was unable to function as intended. An adequately resourced and capacitated health system must be the foundation for a PPP like QMMH for it to operate at the level of a national referral hospital. This would require more balanced investments at all levels of the health system from the beginning of the PPP agreement. Going forward post-PPP, QMMH-IN could support the greater health system by transitioning more fully into a medical training institution focused on building internal and external capacity. This is consistent with recommendations by the WHO [22] as well as those which were generated from the QMMH-IN baseline evaluation [8] and represents a missed opportunity within the QMMH-IN PPP.

### Limitations

This study has several limitations. First the qualitative data explore, but do not attempt to measure the frequency of perspectives and attitudes. Second, interviews were conducted by Boston University researchers, which may have introduced a social desirability bias; however, as evidenced by the quotes, respondents seemed to feel free to express concerns and provide constructive comments on the PPP and QMMH-IN. Third, our sample of 26 included only five respondents from network clinics and thus our findings are likely more hospital-centric in nature. In addition, the scope of this paper did not include perspectives from governmental officials in charge of the regulation and financing of QMMH, nor respondents from district hospitals. As such, the findings as a whole and specifically the recommendations regarding the Ministry of Health’s role/management and greater district health system must be interpreted cautiously.

## Conclusions

Healthcare PPPs are important and promising strategies to finance healthcare systems. It is critical to consider employees as key stakeholders who can help to elicit the nuances of the barriers and facilitators of PPP performance in varying contexts. Implementors of integrative PPPs in lower-resource settings should pay special attention to healthcare system allocation of resources, human resource investment over time, PPP contract flexibility, and succession planning as means of facilitating and sustaining hospital network performance.

## Reflexivity statement

Grounding our methods in reflexivity, here we provide details on authors’ gender, seniority, field of expertise, role in the evaluation, and their relationship to the QMMH-IN PPP. ELNN is a Mosotho while all other study authors are American. BWJ is male while the rest of authors are female. ELNN is a nurse, registered nurse-midwife, holds a Master of Public Health degree and is currently a PhD candidate at Sefako Makgatho University of Health Sciences. CMM and BWJ are family physicians. CMM holds a Master of Science degree in health systems and services research. ELNN, CMM, and BWJ have clinical experience in district hospitals in Lesotho but not within QMMH-IN. All three are also affiliated with Lesotho-Boston Health Alliance, a Lesotho-based organization who served as the implementing partner for the overall evaluation. At the time of data collection, JLK was a Boston University research fellow, with five years of experience in conducting mixed-methods health systems research in sub-Saharan Africa. TN was a Doctor of Public Health candidate at Boston University. JLK and TN, the study interviewers, were not involved in the previous work in Lesotho conducted by Boston University, and held no prior beliefs regarding the performance of QMMH-IN. TV and NAS are both senior public health researchers with a combined 49 years of experience in mixed-methods evaluations.

## Data Accessibility Statement

The qualitative transcripts analyzed for the current study are not publicly available due to the sensitive nature of the end of the PPP and our inability to ensure confidentiality of individual responses. Hospital leadership in particular had unique and public-facing roles and it would not be possible to make their transcripts completely unidentifiable while retaining the content of the information they provided. Please contact the corresponding author with any questions.
